# Application of artificial neural networks to classify *Avena fatua* and *Avena sterilis* based on seed traits: insights from European *Avena* populations primarily from the Balkan Region

**DOI:** 10.1186/s12870-024-05266-3

**Published:** 2024-06-12

**Authors:** Mostafa Oveisi, Danijela Sikuljak, Ana A. Anđelković, Dragana Bozic, Nenad Trkulja, Ramin Piri, Peter Poczai, Sava Vrbnicanin

**Affiliations:** 1 Department of Agronomy and Plant Breeding, College of Agriculture and Natural Resources, University of Tehran, Karaj, Iran; 2 Institute for Plant Protection and Environment, Belgrade, Serbia; 3 Faculty of Agriculture, University of Belgrade, Belgrade, Serbia; 4 Botany and Mycology Unit, Finnish Museum of Natural History, University of Helsinki, Helsinki, Finland

**Keywords:** *Avena fatua*, *Avena sterilis*, Artificial neural networks, Seed traits, Morphological traits, Geographic coordinates, Classification

## Abstract

**Background:**

*Avena fatua* and *A. sterilis* are challenging to distinguish due to their strong similarities. However, Artificial Neural Networks (ANN) can effectively extract patterns and identify these species. We measured seed traits of *Avena* species from 122 locations across the Balkans and from some populations from southern, western, and central Europe (total over 22 000 seeds). The inputs for the ANN model included seed mass, size, color, hairiness, and placement of the awn attachment on the lemma.

**Results:**

The ANN model achieved high classification accuracy for *A. fatua* and *A. sterilis* (R2 > 0.99, RASE < 0.0003) with no misclassification. Incorporating geographic coordinates as inputs also resulted in successful classification (R2 > 0.99, RASE < 0.000001) with no misclassification. This highlights the significant influence of geographic coordinates on the occurrence of *Avena* species. The models revealed hidden relationships between morphological traits that are not easily detectable through traditional statistical methods. For example, seed color can be partially predicted by other seed traits combined with geographic coordinates. When comparing the two species, *A. fatua* predominantly had the lemma attachment point in the upper half, while *A. sterilis* had it in the lower half. *A. sterilis* exhibited slightly longer seeds and hairs than *A. fatua*, while seed hairiness and mass were similar in both species. *A. fatua* populations primarily had brown, light brown, and black colors, while *A. sterilis* populations had black, brown, and yellow colors.

**Conclusions:**

Distinguishing *A. fatua* from *A. sterilis* based solely on individual characteristics is challenging due to their shared traits and considerable variability of traits within each species. However, it is possible to classify these species by combining multiple seed traits. This approach also has significant potential for exploring relationships among different traits that are typically difficult to assess using conventional methods.

**Supplementary Information:**

The online version contains supplementary material available at 10.1186/s12870-024-05266-3.

## Introduction

*Avena fatua* L. (commonly known as wild oat) and *A. sterilis* L. (also known as winter wild oat) are two oat species that belong to the *Poaceae* family and are often found in winter and spring cereals. These species have rapid early growth and establishment, efficient expanded fibrous roots, a high leaf area index, and tall plants, which makes them important competitors in crops [1]. Additionally, they have expanded their presence beyond crop fields and have spread to prairies and urban areas [2]. Although *A. fatua* and *A. sterilis* can grow in different seasons, there are overlaps within the growing seasons, resulting in the coexistence of both species. *A. fatua* is an early spring species, while *A. sterilis* may experience delayed germination due to seed dormancy, age, and physical inhibition by the seed coat [3].

Identifying *A. fatua* and *A. sterilis* is morphologically challenging due to their striking similarities. Both species have counterclockwise leaf orientation and closely resemble each other in terms of ligule, nodes, and small hairs on leaf margins. Identification keys are primarily available during flowering and seed production. Seeds of these species have distinct traits including color, size, hairiness, awn length, awn articulation angle, and awn attachment point on the lemma. Moreover, *A. sterilis* has a longer horseshoe-shaped trace on the seed base, resulting from broken rachillas when compared with *A. fatua*. However, there may be some overlap in the distribution of these traits between the two species, leading to potential misclassifications [4]. Therefore, combining multiple significant traits may provide a more reliable means of distinguishing between these species.

Hybridization between *A. fatua* and *A. sterilis* produces plants with traits that lie between those of the parent species, making them more difficult to identify. These hybrid plants display a mix of characteristics, such as variations in growth habits, leaf morphology, and seed features [5]. The intermediate nature of these traits can complicate their identification, particularly when distinguishing them from the pure parent species or other closely related plants. Accurate recognition and differentiation of these hybrids are essential for effective management strategies, as they can affect crop yield, weed control efforts, and ecological dynamics.

Artificial Neural Networks (ANN) are powerful computational models inspired by the structure and function of the neural networks of the human brain. ANNs imitate the information processing and pattern recognition abilities of biological neural networks. These networks consist of interconnected artificial neurons that communicate and learn from input data to make predictions or classifications [6]. ANNs have been extensively used in plant science due to their ability to analyze complex datasets and extract meaningful patterns [7]. In the context of plant classification, ANNs can be trained to recognize and differentiate plants based on various features, such as seed traits [8]. A model can be trained using a dataset of seed trait measurements. The model learns the patterns and correlations between seed traits and corresponding species labels through a process known as supervised learning. During training, the ANNs adjust their internal weights and biases to minimize the difference between predicted and actual species labels [9]. Once trained, they can classify new, unseen seed samples of *A. fatua* and *A. sterilis*. The seed traits of unknown samples are inputted into the trained ANN, which processes the data through its layers and produces an output prediction indicating the most likely species classification.

In this study, we measured seed traits of *Avena* species from 122 locations primarily in the Balkans and from some populations from southern, western, and central Europe. We analyzed in total over 22 000 seeds. We used morphological seed traits as inputs for the ANN model to investigate the feasibility of classifying *Avena* species. Successful classification of *Avena* species can help overcome the challenges associated with their high morphological similarity. Therefore, our study aimed to determine if there are specific morphological seed traits contributing to each *Avena* species, if these traits are sufficient for accurate classification, and if the seed traits correlate adequately with geographic coordinates, which could facilitate species classification.

## Materials and methods

### Sample collection

**Fig. 1:**
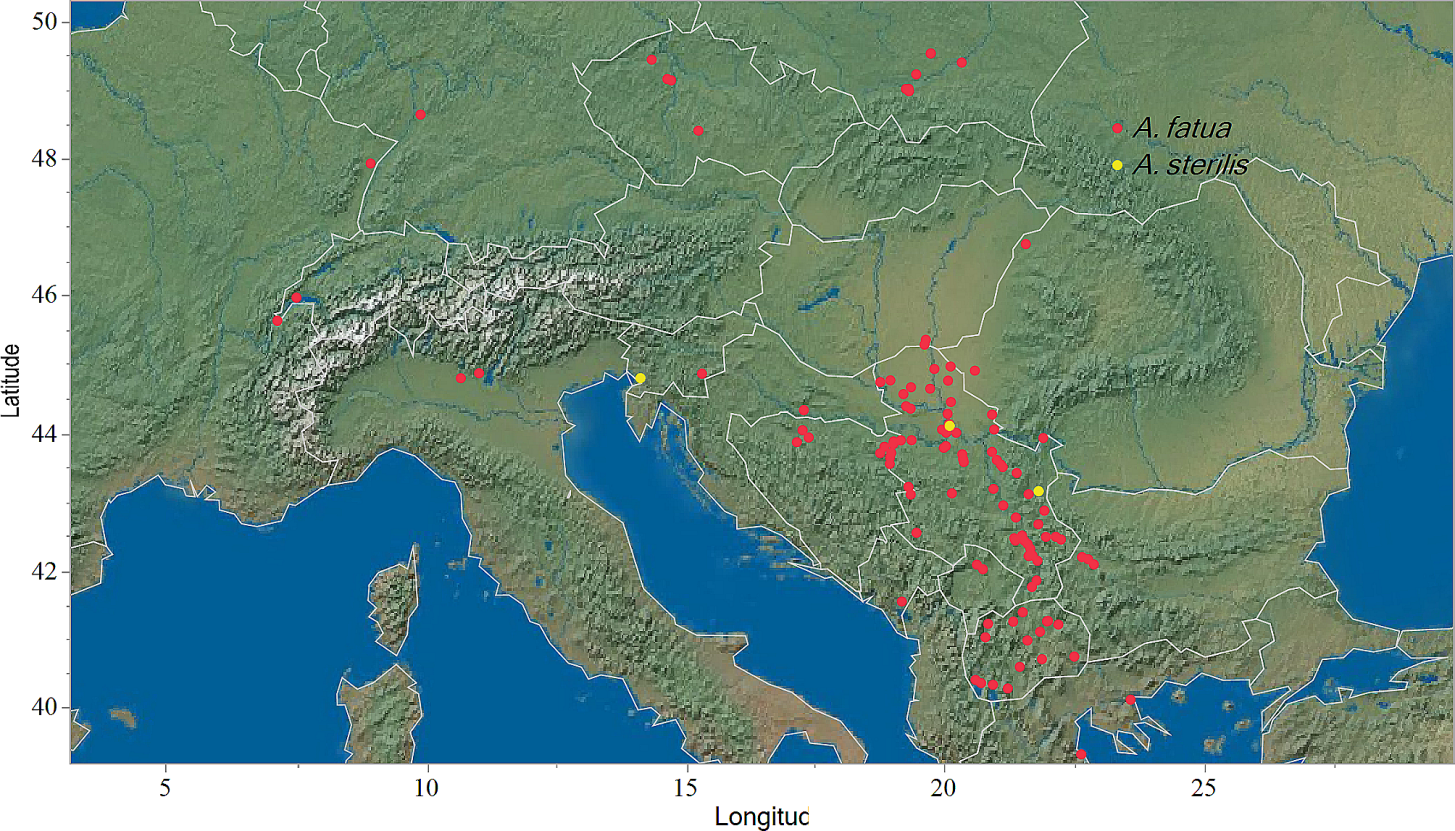
Locations from which *A. fatua* (red points) and *A. sterilis* (yellow points) samples were collected

Samples of *A. fatua* and *A. sterilis* were collected from 122 locations across eastern and western Europe (Fig. 1). In eastern Europe, samples were obtained from 63 locations in Serbia, three in Bulgaria, three in Romania, four in the Czech Republic, and 19 in Poland, Slovenia, and Bosnia and Herzegovina. Additionally, samples were collected from 17 locations in North Macedonia, one in Montenegro, two in Hungary, two in Greece, and one in Croatia. In western Europe, populations were sampled from two locations in Italy, two locations in France, two locations in Switzerland, and one location in Germany. Seed trait measurements were conducted using a total of 180 to 200 seeds from each location, depending on availability. An example of seed morphology is shown in Supplementary Fig. 1.

### Image acquisition

Seed images were obtained using a Stereo Trinocular microscope equipped with a digital camera (model Micro-SC2, EUinstruments). The microscope was set to a 20x magnification and images were captured at a resolution of 1280 × 1024 pixels. To ensure standardized lighting conditions, dual (top and bottom) halogen illumination was used. Prior to imaging, the seeds were placed on a microscopic slide and covered with another slide to ensure proper positioning during the image acquisition process.

### Image analysis

Seed trait measurements were performed using ImageJ version 1.46r (National Institutes of Health, Bethesda, MD, USA). The analysis utilized the following two plugins developed by O’Brien et al. [10]: “Seed Analysis” (version 2.0) and “Color Threshold” (version 1.2). The image analysis in ImageJ involved the following steps: (1) Image Preprocessing, which included background subtraction and noise reduction by applying a Gaussian blur filter with a radius of 2 pixels; (2) Seed Segmentation, where the “Seed Analysis” plugin automatically detected and outlined individual seeds based on their shape and color properties; (3) Seed Measurement, which employed the built-in “Measure” function in ImageJ to obtain measurements of seed length, width, and area; and (4) the “Color Threshold” plugin quantified the intensity of the red, green, and blue channels in the seed images. However, color quality was treated as nominal data for the analysis. Additionally, measurements of hairiness, awn length, awn angle, and awn attachment point were performed on seeds of *A. fatua* and *A. sterilis* using appropriate tools and functions available in ImageJ. The awn attachment point is considered from the base of the seed where it is detached from the rachis, extending towards the apex. Typically, the awn originates from the central third of the seed’s length. If the attachment point is at the midpoint of the seed, it is regarded as 50% of its length; attachment below this point is considered lower than 50%, while attachment closer to the apex is considered higher than 50%.

To quantify the hairiness of seeds, we considered the extent of the area covered by hairs, which are predominantly concentrated at the base of the seeds and extend towards the apex with lower density. This measurement is then converted into a percentage of the total seed area. To convert pixel measurements to physical units, a calibration procedure was conducted using a calibration slide with a known scale (1 mm/100 scale). The calibration factor derived from the calibration slide was applied to all seed measurements to obtain measurements in millimeters.

### Model development

We used a Nominal logistic model to determine the contributions of the measured traits to the nominal data used for classification. Log-worth values and *p*-values were used to compare the effects. Log-worth for each model effect is defined as -log10 (*p*-value). This transformation adjusts *p*-values to provide an appropriate scale for graphing. A value > 2 is significant at the 0.01 level (-log10(0.01) = 2) [11]. Additionally, we conducted a combined stepwise regression analysis to select significant variables for the development of the ANN model.

**Fig. 2:**
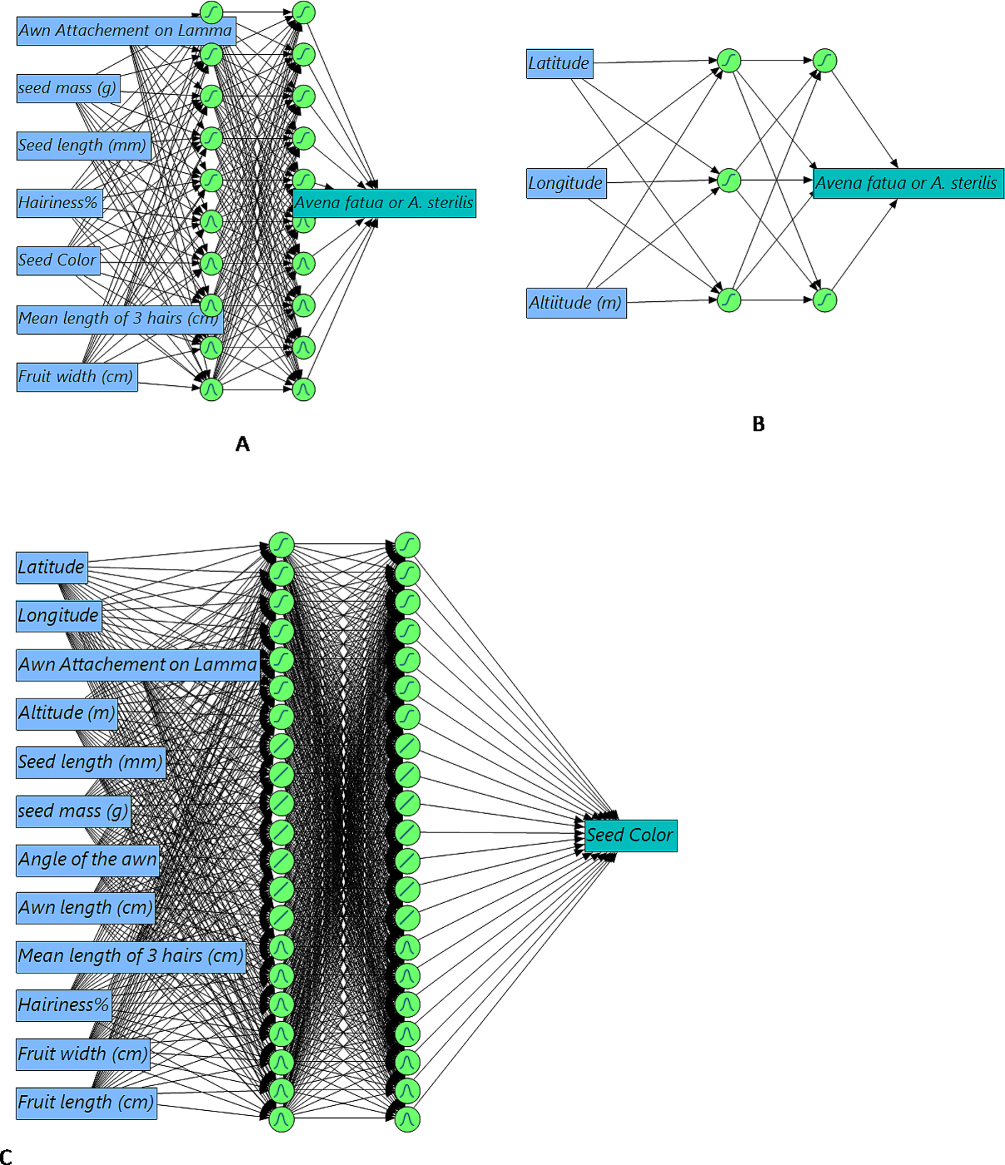
Schematic diagrams indicating model inputs, ANN layers, employed functions, and outputs. (**A**) Diagram for classifying *A. fatua* and *A. sterilis* based on seed traits. (**B**) Diagram for classifying *A. fatua* and *A. sterilis* using geographical data. (**C**) Diagrams for classifying seed color based on seed traits and geographical data

For the classification of *Avena* species using ANNs, the dataset was divided into the following three subsets: 50% for model training, where weights and biases are adjusted; 30% for model validation, which monitors performance during training and prevents overfitting; and 20% for model testing, which evaluates the final model’s performance. The algorithm used to train the ANN was stochastic backpropagation (stochastic gradient descent). The number of training cycles was set as 600 epochs to prevent training from becoming excessive, which could lead to loss of generalization power. The input layer consisted of seven input neurons, which depended on the number of features as predictor variables. The output layer had two neurons for classifying *Avena* species (one for each class). Two hidden layers were also used, each consisting of three hyperbolic tangent functions and three Gaussian functions (Fig. 2). To classify *Avena* species based on geographical coordinates, two hidden layers were employed. The second hidden layer consisted of three hyperbolic tangent functions, while the first layer consisted of two hyperbolic tangent functions (Fig. 2). The input layer included three neurons representing longitude, latitude, and altitude. Similarly, the output layer consisted of two neurons for classifying *A. fatua* and *A. sterilis*. Additionally, ANNs were used for predicting seed color by incorporating both seed traits and geographical coordinates as input variables. For this purpose, each of the two hidden layers employed seven hyperbolic tangents, seven linear, and seven Gaussian functions. To evaluate model performance, besides the generalized R^2^, entropy R^2^, maximum likelihood, and misclassification rate, the matrix of test results was derived from the test data and employed to evaluate the predictive quality of the models. *A. fatua* and *A. sterilis* were classified as negative (N) and positive (P), respectively. The following metrics were calculated using True Positives (TP), False Negatives (FN), True Negatives (TN), and False Positives (FP) rates: Sensitivity = TP/(TP + FN), which measures the proportion of correctly identified actual positive cases (true positives); Specificity = TN/(TN + FP), which measures the proportion of correctly identified actual negative cases (true negatives); Accuracy = (TP + TN)/(TP + TN + FP + FN), which provides an overall measure of the model’s performance across all classes; F1-score = 2*TP/(2TP + FP + FN), which balances precision and recall (sensitivity) into a single metric; and Matthews Correlation Coefficient (MCC) = (TP*TN-FP*FN)/sqrt((TP + FP)*(TP + FN)*(TN + FP)*(TN + FN)), which is a comprehensive metric considering TP, TN, FP, and FN. MCC ranges from − 1 (perfect disagreement) to + 1 (perfect agreement).

Principal Component Analysis (PCA) was also used to explore the correlations among *Avena* seed traits and geographical coordinates. Various principal component (PC) combinations were assessed for explaining variances using a scree plot and eigen values in addition to the biplot. Finally, PC1 and PC2 were chosen for visualization and interpretations.

Data analysis and graphing were performed using JMP® Version 17.1, SAS Institute Inc., Cary, NC, 1989–2023.

## Results

### Application of artificial neural network models

Nominal logistic analysis revealed that the awn attachment point on the lemma, seed length, and seed color had the lowest *p*-values and the highest log-worth values (160.7, 96.4, and 28.8, respectively) (Table 1). This suggests that these traits are the most significant distinguishing characteristics between *A. fatua* and *A. sterilis*. Hairiness, seed mass, and mean length of three hairs were ranked as the second and third most important traits (all *p* < 0.06). Fruit width, length, awn length, and the angle of awn were not significant factors in distinguishing between *A. fatua* and *A. sterilis*. Therefore, based on the results of the Nominal logistic analysis and combined stepwise analysis, we selected seed mass, awn attachment point on the lemma, seed length, seed color, hairiness, fruit width, and mean length of three hairs as inputs for the ANN model (Fig. 2).

**Table 1:** Contribution of seed traits in classifying A. fatua and A. sterilis using nominal logistic analysis

Avena seed measures	Log-worth	*P*-value
Awn attachment point on lemma	160.76	0.000001
Seed length (mm)	96.42	0.000001
Seed color	28.88	0.000001
Hairiness (%)	2.52	0.0088
Seed mass (g)	1.57	0.027
Mean length of 3 hairs (cm)	1.2	0.064
Fruit width	0.64	0.22
Angle of the awn	0.47	0.34
Awn length (cm)	0.28	0.52
Fruit length (cm)	0.056	0.88

The performance of the ANN model was evaluated during the training, validation, and testing stages. The model exhibited exceptional performance, generating generalized and entropy R^2^ values of 0.99 and a RASE < 0.0003. Misclassification rates remained consistently at zero throughout the training, validation, and testing phases (Table 2). Moreover, the values of sensitivity, specificity, accuracy, F1-score, and MCC validate the strong performance of the model (Table 3). These findings demonstrate that differentiating between *A. fatua* and *A. sterilis* based on their seed morphological traits can be effectively accomplished using an ANN model.

**Table 2:** Performance of artificial neural network models in classifying A. fatua and A. sterilis using seed traits, geographical data, and classifying seed colors using seed traits and geographical data

Measures	Training			Validation					Test		
	Avena Classification (Seed traits)	Avena Classification (Geographic coordinates)	Seed color Classification	Avena Classification (Seed traits)		Avena Classification (Geographic coordinates)		Seed color Classification	Avena Classification (Seed traits)	Avena Classification (Geographic coordinates)	Seed color Classification
Generalized R^2^	0.99	0.99	0.92	0.96	0.99		0.86		0.99	0.99	0.87
Entropy R^2^	0.99	0.99	0.81	0.96	0.99		0.73		0.99	0.99	0.75
RASE	0.0009	0.0002	0.20	0.015	0.0002		0.26		0.0005	0.0002	0.21
Mean Abs Dev	0.000003	0.00002	0.13	0.0003	0.00002		0.19		0.00002	0.000002	0.17
Misclassification Rate	0	0	0.11	0.0022	0		0.17		0	0	0.15
Sum Freq	12,460			7516					4632		

**Table 3:** Metrics for evaluating ANNs obtained from confusion matrix of test data

Model	Sensitivity	Specificity	Accuracy	F1-score	MCC
A	1	1	1	1	1
B	0.97	0.96	0.96	0.97	0.96
C	0.82	0.81	0.83	0.81	0.82

**A:** Classifying *A. fatua* and *A. sterilis* based on seed traits; **B:** Classifying *A. fatua* and *A. sterilis* based on geographical data; **C:** Classifying seed colors based on seed traits and geographical data

**Table 4:** Seed traits and geographical data contribution in classifying seed colors using nominal logistic analysis

Source	Log-worth	*P*-Value	
Seed mass (g)	162.028	0.00001	
Fruit width (cm)	138.324	0.00001	
Longitude	86.915	0.00001	
Awn Attachment on Lemma	65.149	0.00001	
Hairiness (%)	45.887	0.00001	
Seed length (mm)	44.937	0.00001	
Altitude	29.178	0.00001	
Latitude	20.806	0.00001	
Angle of the awn	16.814	0.00001	
Mean length of 3 hairs (cm)	12.685	0.00001	
Fruit length (cm)	0.626	0.23636	
Awn length (cm)	0.095	0.80380	

### Comparing seed traits between *A. fatua* and *A. sterilis*

**Fig. 3:**
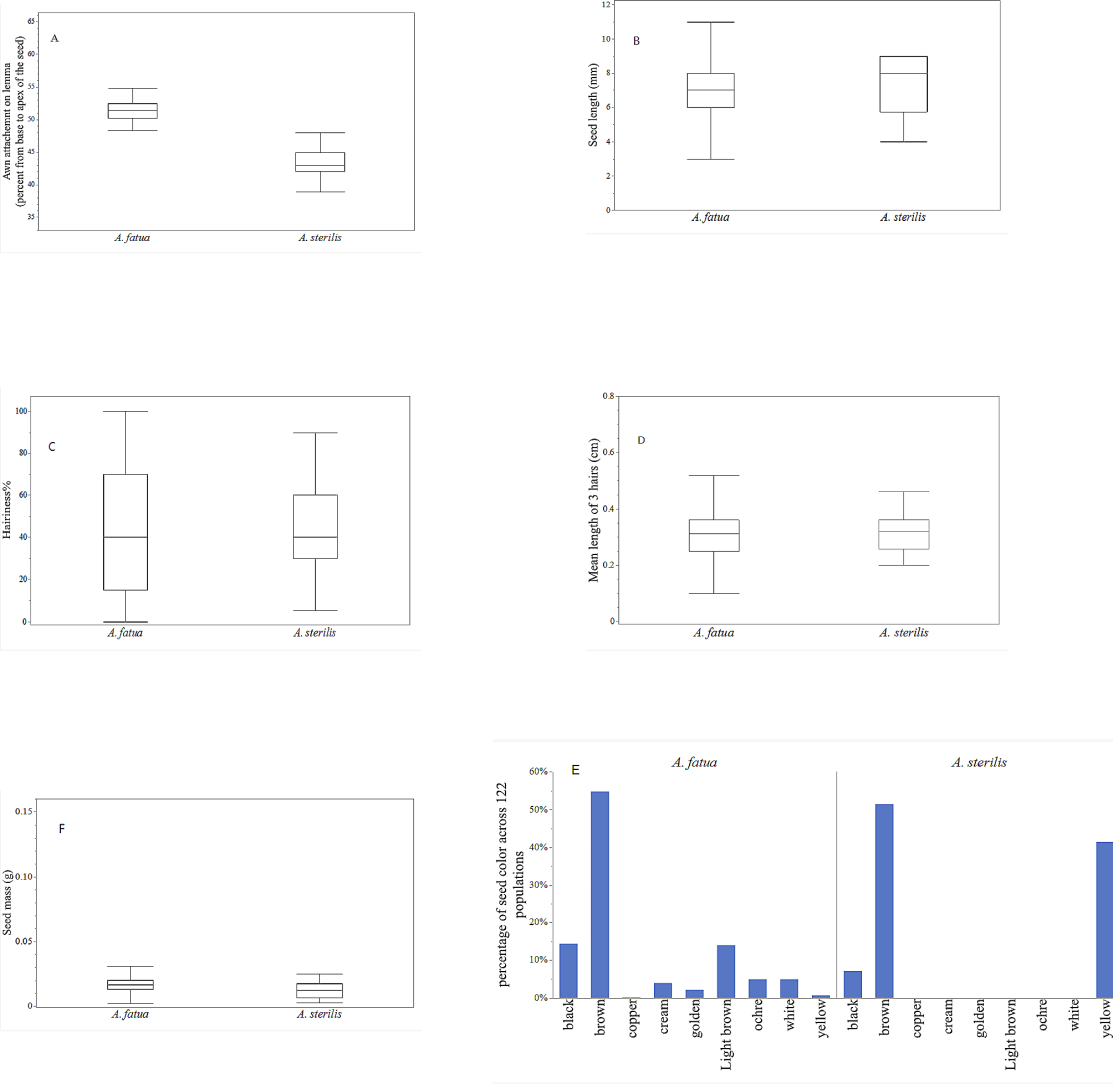
Comparison of morphological seed traits (**A** to **F**) used as inputs for the classification of *A. fatua* and *A. sterilis*. The seed traits are compared between *A. fatua* and *A. sterilis*

We used the ANN model (Fig. 3) to compare the morphological seed traits of *A. fatua* and *A. sterilis*. The attachment points of the awn on the lemma showed high variability, which was a distinguishing trait between the two species. In *A. fatua*, the attachment point is mostly located along the upper half of the lemma, whereas in *A. sterilis*, it is positioned along the lower half (Fig. 3, A). *A. sterilis* had a slightly longer average seed length, but this difference was not statistically significant between the species (Fig. 3, B). Seed hairiness also varied when comparing the two species (Fig. 3, C) and did not differ significantly between *A. fatua* and *A. sterilis*. However, this trait played a significant role in classifying *Avena* species. When comparing the mean length of three hairs, *A. sterilis* exhibited lower variability and, on average, longer hairs than *A. fatua* (Fig. 3, D). Conversely, *A. fatua* showed high variability in this trait. We found no evidence of a difference in seed mass between *A. fatua* and *A. sterilis* (Fig. 3, F). Seed color was identified as an important trait contributing to the classification of *Avena* species. In *A. fatua* populations, brown seeds were the most prevalent, followed by light brown and black seeds (Fig. 3, E). Copper- and yellow-colored seeds were less common. Fewer variations in seed color were observed in *A. sterilis* populations, which had smaller populations. The observed seed colors in *A. sterilis* were black, brown, and yellow.

**Fig. 4:**
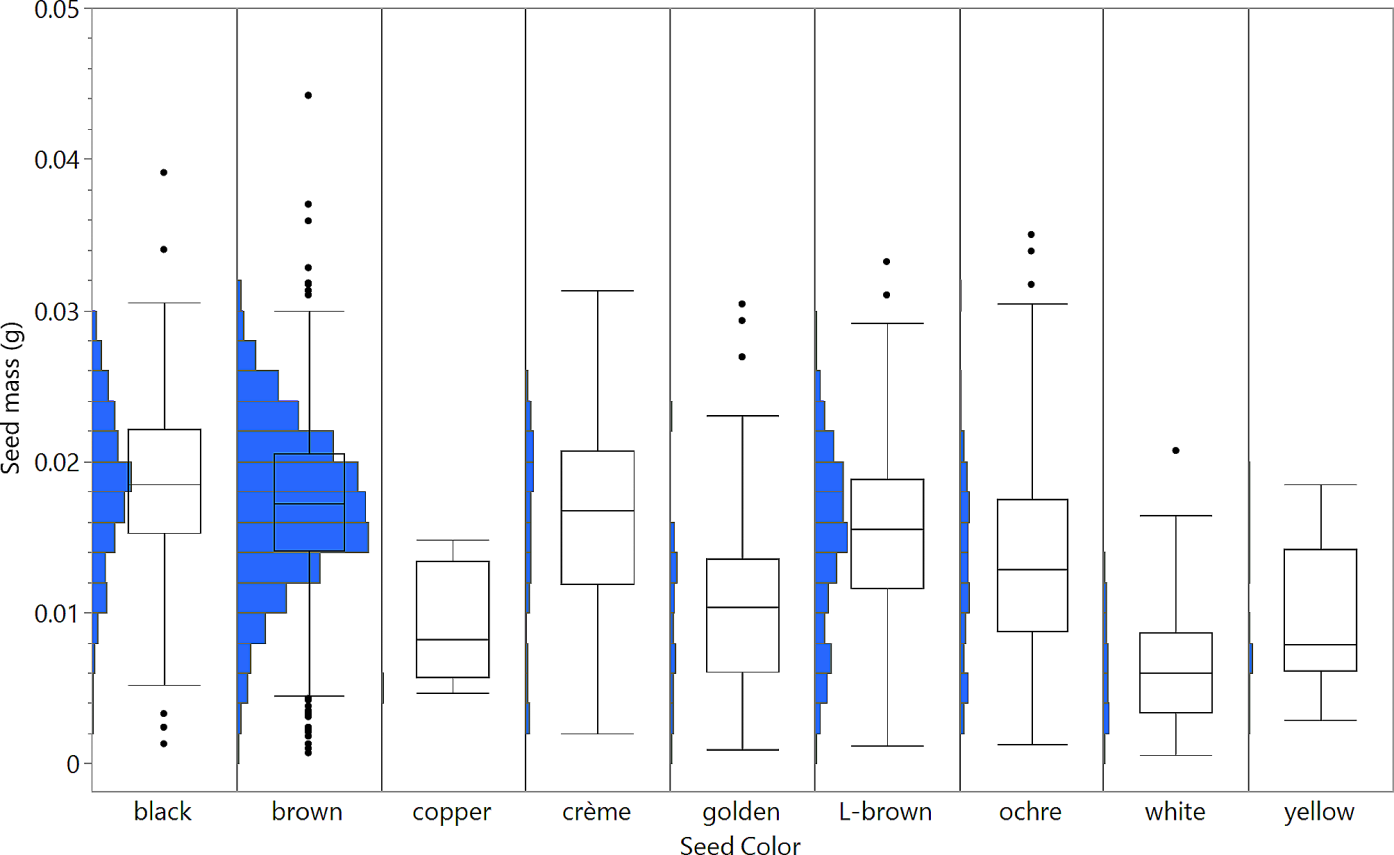
Abundance and distribution of seed color and seed mass within *Avena* populations

Although there were interactions between some morphological traits, they were not statistically significant due to the large variations observed across populations. However, a significant interaction was observed between seed mass and seed color (Fig. 4). Seeds with black and brown colors, which were the most frequent, had the highest mean seed mass. On the other hand, seeds with white, yellow, and copper colors, which had the lowest frequencies, had the lowest average seed mass.

### Geographical coordinates data determine the distribution of *A. fatua* and *A. sterilis*

The geographical coordinates of the sampling locations were an important variable in classifying the sampled *Avena* species. We used a two-layered ANN model with three hyperbolic tangent functions at each layer (Fig. 2) to efficiently classify *A. fatua* and *A. sterilis*. No misclassifications were observed during the training, validation, and testing phases. The general and entropy R^2^ values were > 0.99, and the RASE values were < 0.00001 for all training, validation, and testing sets (Table 2). This aligns with sensitivity, specificity, accuracy, F1-score, and MCC values greater than 0.9, confirming the performance of ANNs in discriminating *A. fatua* and *A. sterilis* using geographical data (Table 3). Therefore, the geographical coordinates of the study areas were a crucial factor in accurately classifying the two *Avena* species.

**Fig. 5:**
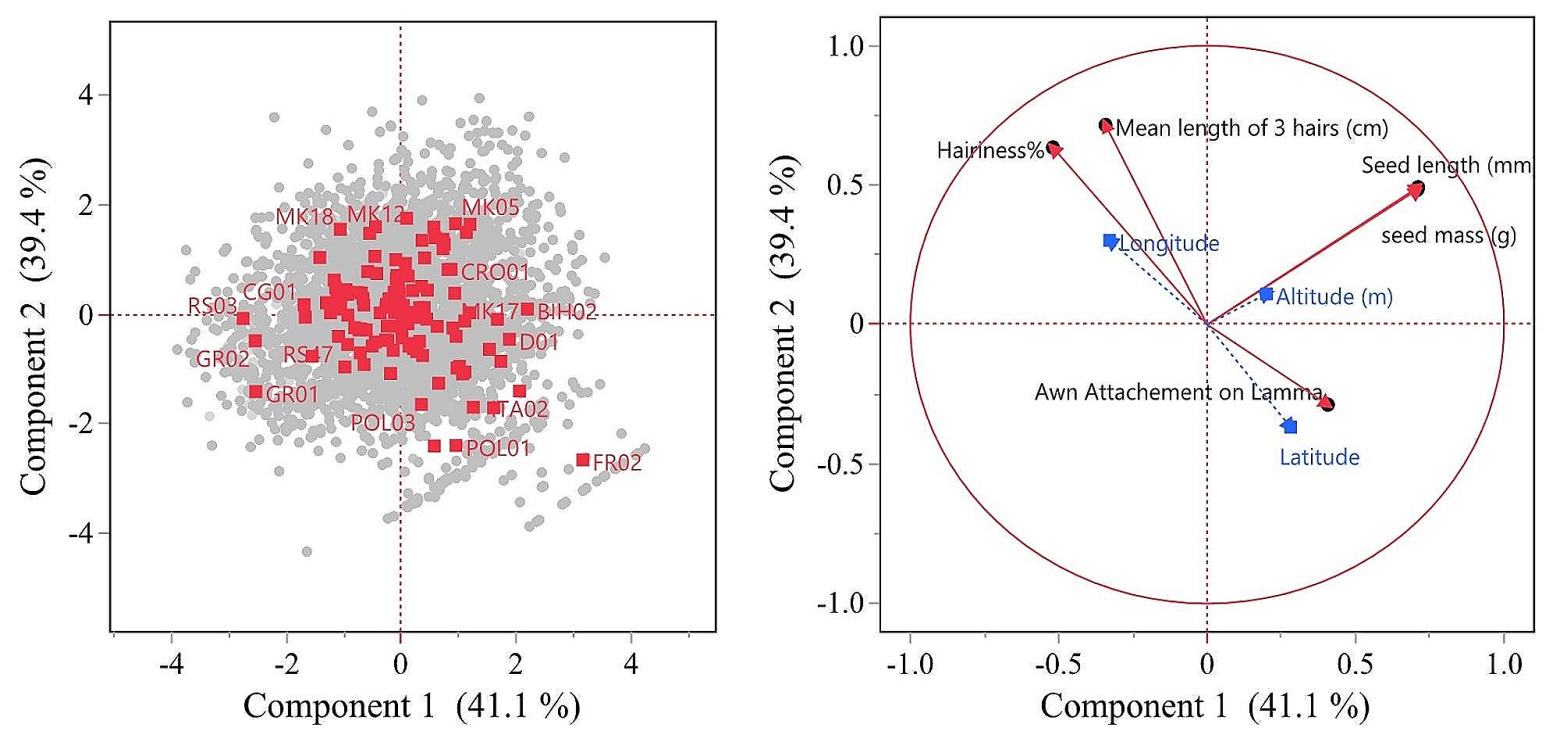
Biplot obtained from PCA analysis showing relationships among seed traits, geographical data, and *Avena* species. Components 1 and 2, which account for 80.5% of the variations, are shown

*A. sterilis* was primarily found at altitudes below 400 m, while *A. fatua* was found in areas above 400 m (Fig. 1). The biplot (Fig. 5) from the PCA revealed a strong positive correlation between seed mass, seed length, and altitude. Hairiness, hair length, and longitude also exhibited a positive correlation, while the awn attachment on the lemma correlated with latitude. Additionally, there was a negative correlation between awn attachment on the lemma and hairiness and hair length. Thus, seeds with awns attached to the lower half of the lemma, along with higher hairiness and hair length, indicated greater hairiness of *A. sterilis* seeds.

**Fig. 6:**
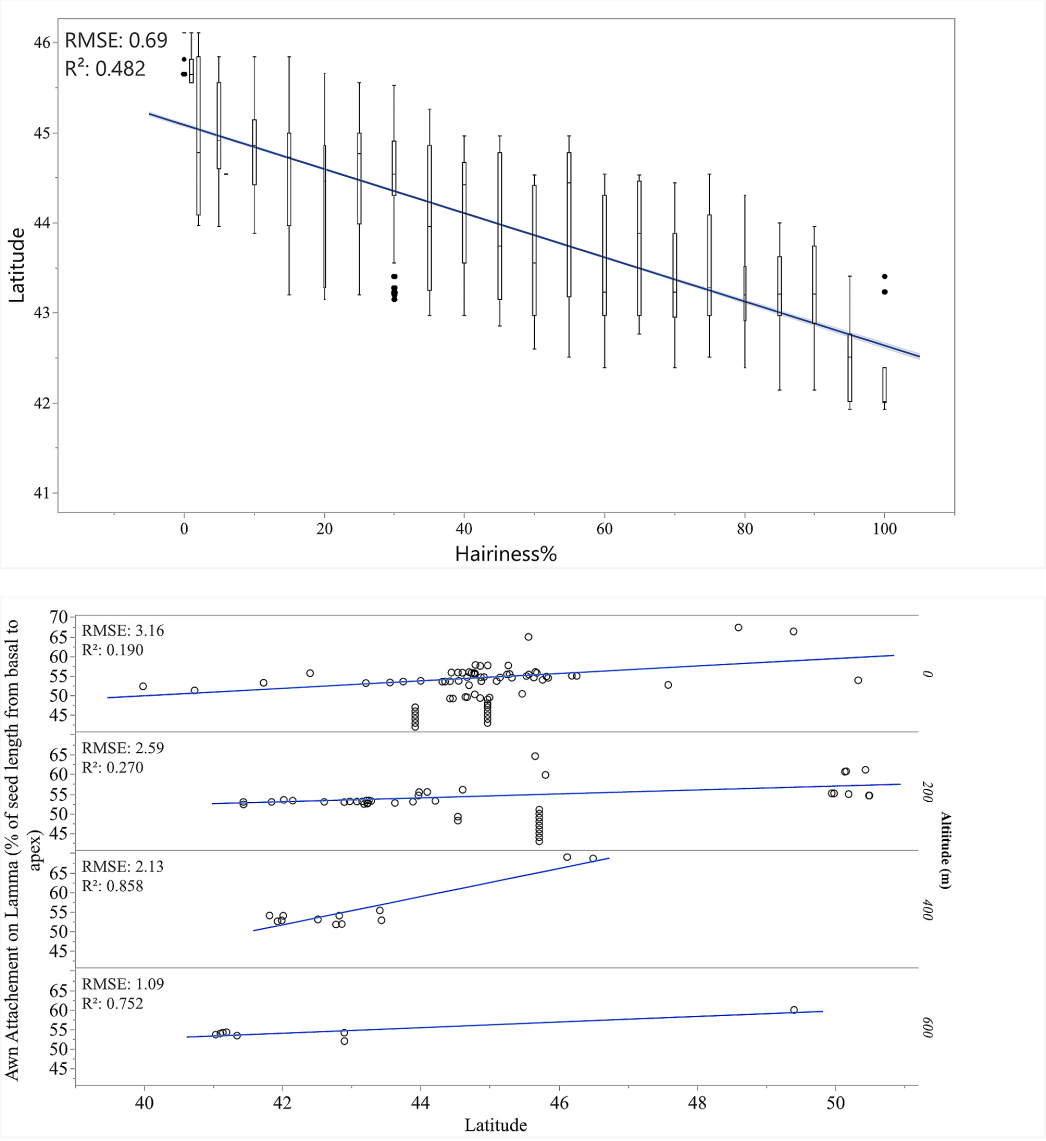
Variations in hairiness percentage of seeds with changing latitude (**A**) and awn attachment point on lemma as a percentage from basal to apex with changing latitude and altitude (**B**)

While examining the potential relationship between seed traits and geographical coordinates, we noticed a decrease in seed hairiness as latitude increased (Fig. 6). Fully hairy seeds (100%) were mostly found at lower latitudes (42–43), while less hairy to hairless seeds were more common at higher latitudes (45–46). Additionally, when studying the change in awn attachment point on the lemma with latitude, we observed a general trend towards the upper half of the seeds as latitude increased (Fig. 6). Interestingly, this response seemed to be influenced by the altitude of the sampling locations. When analyzing the awn attachment point on the lemma in relation to both latitude and altitude, the shift towards the upper half of the lemma became more significant at higher altitudes, particularly those above 400 m.

### Predicting seed color using seed traits and geographic coordinates

Nominal logistic analysis revealed significant effects of longitude, latitude, and altitude on variations in seed color. In addition, all seed traits, except for awn and fruit length, significantly contributed to determining seed color (Table 4). Using ANNs, we primarily attempted seed color classification based on seed traits without including geographical variables in the input neurons. However, its best prediction was not better than an R^2^ of 0.67, RASE of 0.51, and a misclassification rate of 0.3. The confusion matrix demonstrated misclassification of colors, especially for those colors that were less frequent. Therefore, an ANN model with two layers incorporating logistic, Gaussian, and linear functions was used to classify seed colors based on both seed traits and geographic coordinates as the input neurons (Fig. 2). The results showed that seed colors can be predicted to a high extent based on seed traits and geographic coordinates, although with less accuracy compared to *Avena* species classification. The R^2^ values of 0.92 (training set), 0.86 (validation set), and 0.87 (testing set), the misclassification rates of 0.13 (training set), 0.19 (validation set), and 0.17 (testing set) (Table 2), and the metric values from the confusion matrix of the test data were also greater than 0.8 (Table 3), all indicating the potential of seed color classification.

## Discussion

### Performance of ANNs in discriminating between *A. fatua* and *A. sterilis* seeds

ANNs are frequently used to discriminate between similar objects in the field of plant science, such as distinguishing between weeds and crops [12] or to discriminate between different plant varieties within the same genus [13, 14]. They have also been used to classify plant leaves and detect diseases in tomato plants [15], to detect yellow rust and nitrogen deficiency in wheat [16], or to classify potato plants infected with potato virus Y [17]. ANNs have also been successful in seed classifications, such as discriminating between seeds from green, orange, red, and yellow pepper cultivars [18], sorting wheat seeds [19], and grading rice seed quality [20]. Despite the application of ANNs in various fields where classification of individuals of close resemblance is of high interest, neither ANNs nor any machine-learning methods have been used to classify *A. fatua* or *A. sterilis*. Discriminating between these two species has been a significant challenge for research and practical management, a challenge that has been addressed in this study. We demonstrated that there are specific seed traits that can accurately determine the species to which a seed belongs. This highlights the high usefulness of seed appearance data in distinguishing between *A. fatua* and *A. sterilis* seeds, particularly when their mixed presence is crucial.

### Contribution of seed characteristics in distinguishing *Avena fatua* and *Avena sterilis*

Distinguishing between *A. fatua* and *A. sterilis* can be challenging due to their shared similarities at different growth stages. However, our study has identified key characteristics that effectively differentiate the seeds of these closely related species.

#### Positioning of the awn attachment on the lemma

Our investigation revealed that the point where the awn attaches to the lemma is a crucial factor in distinguishing between *A. fatua* and *A. sterilis*. Through nominal logistic analysis, we determined that the awn attachment had the highest log-worth values, highlighting its significance in classifying the species. Awns in the Gramineae family are positioned variably, either at the tip (apically) or at a point on the back of the lemma (abaxially) [21]. Specifically in the study species, the awn is attached to the middle one-third portion of the lemma. Attachments in the upper half correspond to *A. fatua*, while those in the lower half correspond to *A. sterilis*. Upper attachment in *A. fatua* promotes wind dispersal, as it allows the seed to catch wind currents closer to the apex, aiding colonization. Lower attachment in *A. sterilis* facilitates seed burial in the soil, enhancing water absorption during periods of scarcity [22, 23]. Consistent with our results, some references mention the awn characteristics as a key feature for distinguishing between *A. fatua* and *A. sterilis* [4]. However, these references do not specifically point out the different position of awn attachment as a key factor for discriminating between these two species.

#### Seed length and mass

Seed length and mass are important features for distinguishing between species. *A. sterilis* had a slightly greater mean seed length than *A. fatua*, while mean seed mass was not different. However, both species exhibited high variability in these traits, making them unreliable for distinguishing between *A. fatua* and *A. sterilis*. Wu et al. [24] suggested a positive correlation between seed mass and altitude within species. Our study also found a strong correlation between seed length and mass with latitude and altitude. However, conflicting reports indicate a decrease in seed mass and length as altitude increases [25]. Therefore, while seed mass and seed length can be used in combination with other traits to differentiate *Avena* species, they should not be relied upon exclusively. Additionally, the length of the first floret in *A. sterilis* ranges from 15.0 to 35.0 mm, while the second floret is 10.0 to 14.0 mm [26]. Seed length of the second floret in *A. sterilis* overlaps with the seed length range of the first floret in *A. fatua*. This overlap can be confusing during field assessments.

#### Seed hairiness

The average hairiness of seeds and length of seed hairs did not differ significantly between *A. fatua* and *A. sterilis*. However, these factors played a significant role in distinguishing between the two species using ANNs. Trichome density, or seed hairiness, provides significant benefits to plants. It acts as a physical barrier, protecting seeds from excessive UV radiation, wind, insects, and pathogens [27]. Our findings indicate a positive correlation between seed hairiness and longitude, while a negative correlation was observed with latitude. Lower latitudes are associated with a higher percentage of hairiness on the seed surface. Therefore, seed hairiness may vary when samples are collected from different geographical coordinates [28].

#### Seed color

Genetic factors play a crucial role in determining seed color and can sometimes be used as a basis for identifying genotypes [29]. We have shown that seed color can be predicted by other seed traits, such as seed length, hairiness, and awn characteristics. However, the rate of misclassification was high, which means that using seed color has potential but is not yet reliable. Seed color was formerly considered to be a size-related trait associated with seed mass, length, or width [30]. Immature seeds are generally smaller and display different colors compared to fully mature seeds. During seed development, there is usually an increase in size accompanied by potential color changes [31]. Seed mass is influenced by the nutrient reserves stored within the seed to support the developing embryo until it can sustain itself through photosynthesis. Seed color can indicate the presence of pigments or compounds related to nutrient storage and environmental protection [32]. Certain genes may regulate both traits simultaneously or have interconnected effects. For instance, genes involved in pigment production can impact seed mass through shared metabolic pathways or hormonal regulation. The hairs on the seed surface can also be associated with seed color due to pigment deposition. The hairs may contain pigments, such as anthocyanins or carotenoids, which contribute to colors such as red, orange, yellow, or brown. These pigmented hairs contribute to the overall color and visual appearance of the seed [33]. Furthermore, seed hairs can create a color contrast with the seed surface, making it more visible and attractive to seed dispersers [34]. However, inconsistencies in seed color within an individual inflorescence or plant [35] and environmental factors [36], which were also consistently shown in this study, may create ambiguity when using seed color as a key for species classification.

Plant coloration can vary significantly based on geographic coordinates, primarily due to abiotic factors that change across latitudes or longitudes. When distinguishing between *A. fatua* and *A. sterilis* based on color, it is crucial to differentiate them within the same geographic area in which they often coexist. However, seed color within a species is not consistent even within the same region. Additionally, both *A. fatua* and *A. sterilis* share similar seed colors in the same area, suggesting that seed color as an individual characteristic for distinguishing species may not be reliable.

Hoffmann and Sgrò [37] reported that *A. fatua* exhibits variation in floret and lemma color. The floret is generally shiny reddish-brown with a straw yellow pointed end, but can also appear straw yellow, dark brown, or gray. In another study, Nečajeva et al. [38] found that within the same collection area of *A. fatua*, lemma color varied between ocher, light-brown, brown, and black. Brown lemma color was predominant in most analyzed populations. Our own sampling also revealed various colors within the same area.

Furthermore, when we incorporated geographic coordinates and altitude as input variables, ANNs performed better in predicting seed color. This finding aligns with a previous study [39] that demonstrated how seed color is influenced by geographic factors. Therefore, seed color lacks consistency across changing environments.

## Conclusions

We have identified potential distinguishing features in the seeds of *A. fatua* and *A. sterilis*. The placement of awn attachment on the lemma can be used to classify species. Although the awn attachment on the lemma exhibits variability within populations of the same species across different geographical coordinates, the variation between species remains distinct compared with the variations within species. *A. fatua* attached to the upper half of the lemma while *A. sterilis* attached to the lower half, providing a consistent measure to distinguish between the two species. However, most seed traits, including length, mass, hairiness, and color, significantly overlap. Therefore, it is necessary to examine these traits in combination for successful classification. Additionally, geographical coordinates may significantly influence seed features. Thus, there are inconsistencies in important seed traits, such as mass, size, color, or hairiness, due to changing geographical coordinates from which seed samples were collected. To achieve a more thorough classification, it would be advantageous to broaden the study by including samples from a wider range of environments. This would enhance the accuracy in attributing highly variable traits, such as seed color or size, to a specific species. Furthermore, there are other spikelet-related characteristics that would assist in expanding the use of ANNs to a broader range of seed traits.

### Electronic supplementary material

Below is the link to the electronic supplementary material.

**Supplementary Material 1**: **Supplementary Fig. 1**. Example of seeds with sampling codes used in our study for ANN models.

## Data Availability

The data supporting the findings of this study are available upon formal request. Researchers interested in accessing the data should contact the corresponding author, Sava Vrbnicanin, at sava@agrif.bg.ac.rs. To initiate the request, please provide a detailed description of the purpose for which the data are needed. Access will be granted based on adherence to ethical standards, privacy regulations, and compliance with any relevant institutional policies. Requests will undergo a thorough review by the research team, and access may be granted at their discretion.

## Seed material identification

Formal identification of the seed material used in the study was conducted by Sava Vrbnicanin and Danijela Sikuljak. While no specimens have been deposited in an herbarium, all analyzed samples have been securely stored in the publicly accessible weed seed collection of the Department of Pesticides and Weed Science at the Faculty of Agriculture, University of Belgrade, Serbia. These samples are available for review upon request.
