# Supplementary material for: Application of artificial neural networks to classify Avena fatua and Avena sterilis based on seed traits: insights from European Avena populations primarily from the Balkan Region
Source: BMC Plant Biol. 2024 Jun 12;24:537. doi: 10.1186/s12870-024-05266-3 (PMC11167764; doi:10.1186/s12870-024-05266-3)
Supplement: Supplementary file 1 — Supplementary Material 1: Supplementary Fig. 1. Example of seeds with sampling codes used in our study for ANN models. [file 12870_2024_5266_MOESM1_ESM.pptx]

## Slide 1
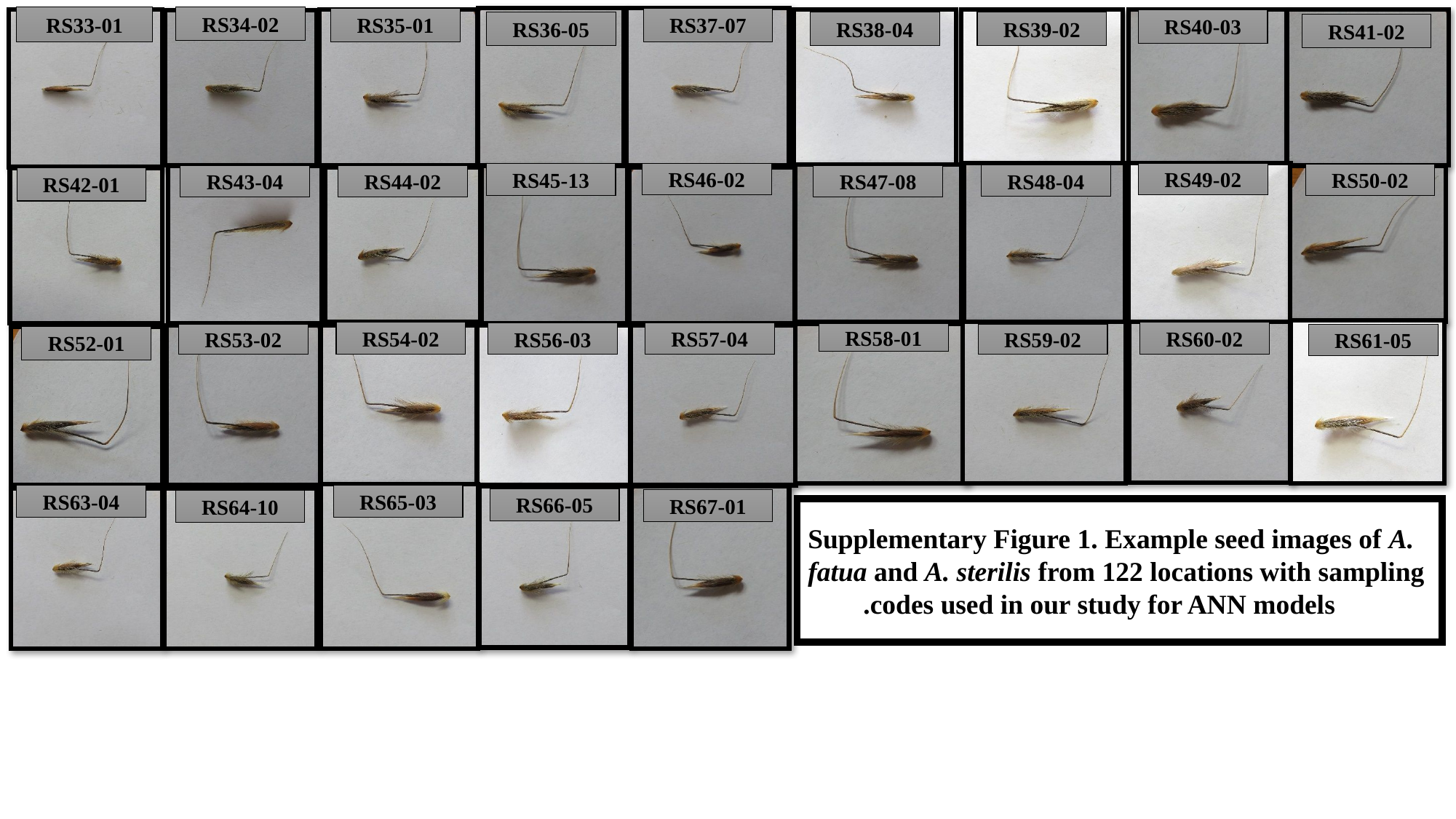

RS33-01
RS34-02
RS35-01
RS37-07
RS40-03
RS38-04
RS39-02
RS36-05
RS41-02
RS46-02
RS49-02
RS45-13
RS50-02
RS48-04
RS44-02
RS47-08
RS43-04
RS42-01
RS54-02
RS60-02
RS57-04
RS56-03
RS58-01
RS61-05
RS53-02
RS59-02
RS52-01
RS65-03
RS63-04
RS66-05
RS67-01
RS64-10
Supplementary Figure 1. Example seed images of A. fatua and A. sterilis from 122 locations with sampling codes used in our study for ANN models.

## Slide 2
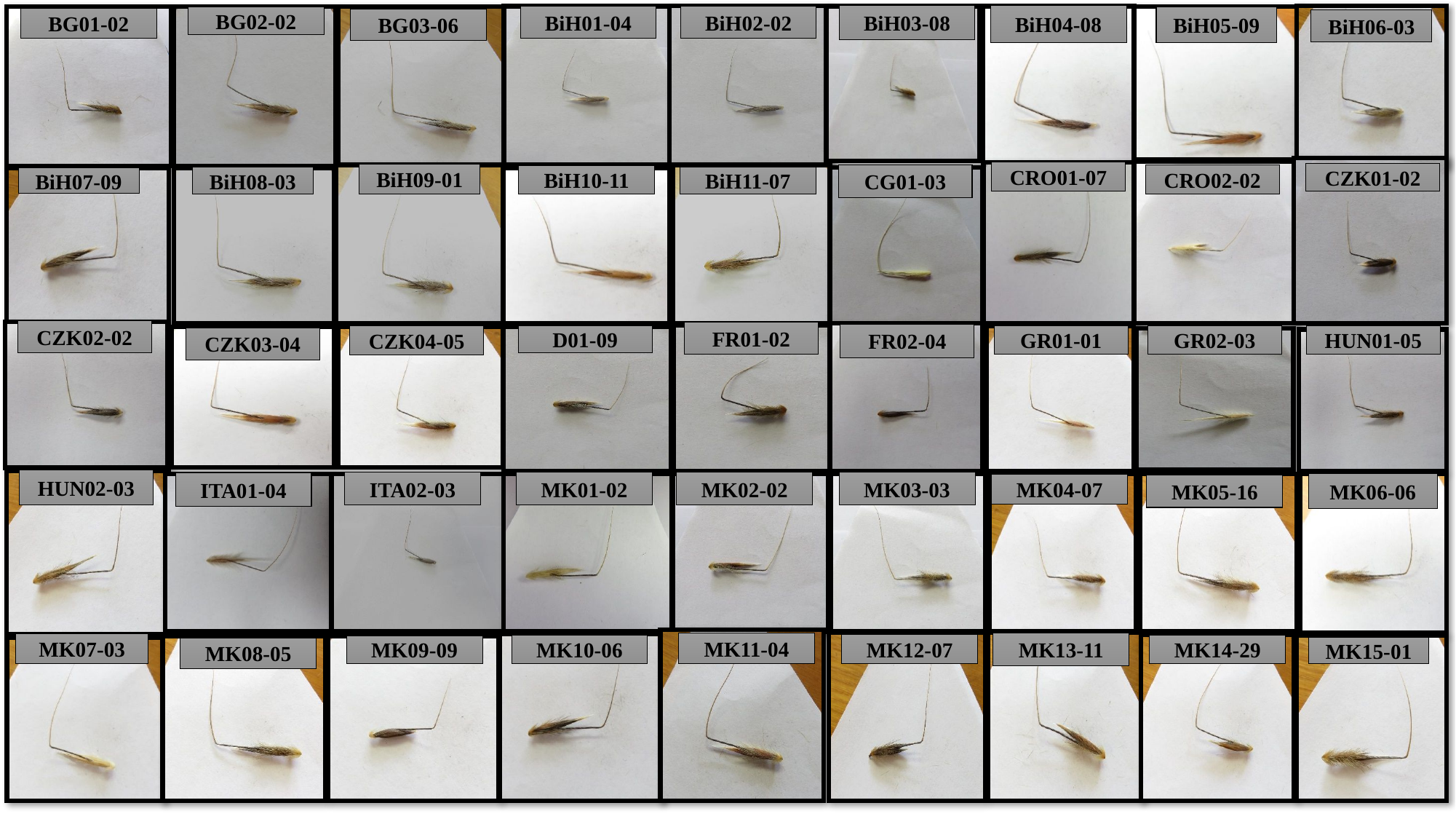

BiH03-08
BiH01-04
BiH02-02
BG02-02
BG01-02
BG03-06
BiH04-08
BiH05-09
BiH06-03
BiH09-01
BiH08-03
BiH07-09
BiH10-11
BiH11-07
CRO01-07
CRO02-02
CG01-03
CZK01-02
CZK02-02
CZK04-05
CZK03-04
FR01-02
FR02-04
D01-09
GR01-01
GR02-03
HUN01-05
HUN02-03
MK01-02
MK03-03
ITA02-03
MK02-02
ITA01-04
MK06-06
MK04-07
MK05-16
MK13-11
MK11-04
MK12-07
MK10-06
MK14-29
MK09-09
MK15-01
MK08-05
MK07-03

## Slide 3
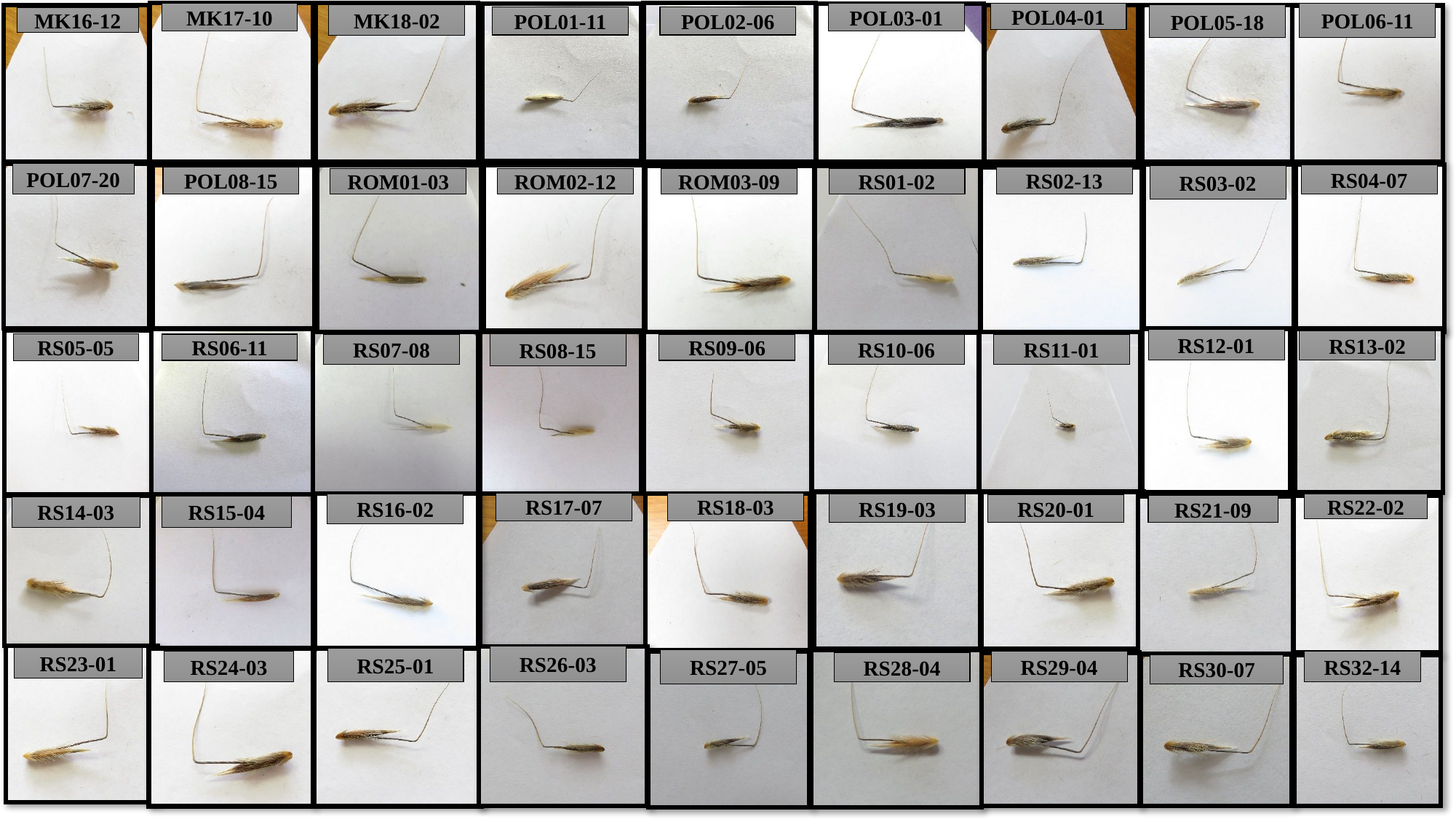

POL03-01
POL06-11
POL04-01
MK17-10
MK18-02
POL05-18
POL01-11
POL02-06
MK16-12
POL07-20
RS04-07
RS03-02
RS02-13
POL08-15
ROM01-03
ROM02-12
ROM03-09
RS01-02
RS12-01
RS13-02
RS10-06
RS11-01
RS06-11
RS07-08
RS08-15
RS09-06
RS05-05
RS18-03
RS16-02
RS19-03
RS22-02
RS20-01
RS21-09
RS15-04
RS17-07
RS14-03
RS26-03
RS25-01
RS27-05
RS24-03
RS29-04
RS32-14
RS28-04
RS30-07
RS23-01
